# Cure for Hiccups

**Published:** 1849-05

**Authors:** 


*Cure for Hiccups.*—Traveling some time since, by rail-road, from Columbus to Baltimore, I took my seat immediately in front of a gentleman who was suffering under a paroxysm of hiccup, to a degree that I had never before witnessed. In a few minutes a person appeared from the end of the car, and took a seat beside him, when he said, 'Sir, can you tell me what is good for the hiccups? I have been afflicted in the way you see me, since yesterday noon, and have had no rest, or relief from a physician to whom I applied for assistance; I am worn out with suffering.' To whom the person replied, 'Sir, I will cure it in less than two minutes, by your watch; have confidence, for I am sure I can do it. Hold up, high, above your head, two fingers of your hand; lean back in your seat, opening your mouth and throat, so as to give free passage to your lungs; breathe very long and softly, and look very steadily at your fingers.' In less than the time specified, the cure was performed, one hiccup only occurring during the trial. The patient could not express his gratitude, while the practitioner only exacted from him, as a fee, the promise that he would extend the knowledge which he had imparted, as freely as he had received it, assuring him that he would never be disappointed in the result.

We were all struck with the fact, and many of us considered that the stranger was sent by the appointment of that Power, often designated as a *particular Providence*. Since then, I have often had occasion to practice upon patients, in the same disorder, and never without the most signal success.—*Southern Med. and Surg. Journal.*

